# Toward equitable open research: stakeholder co-created recommendations for research institutions, funders and researchers

**DOI:** 10.1098/rsos.221460

**Published:** 2023-02-01

**Authors:** Nicki Lisa Cole, Stefan Reichmann, Tony Ross-Hellauer

**Affiliations:** ^1^ Open and Reproducible Research Group, Graz University of Technology, Graz, Austria; ^2^ Know-Center GmbH, Graz, Austria

**Keywords:** open science, open research, open science policy, open science recommendations, equity in science, meta-science

## Abstract

Open Research aims to make research more accessible, transparent, reproducible, shared and collaborative. Doing so is meant to democratize and diversify access to knowledge and knowledge production, and ensure that research is useful outside of academic contexts. Increasing equity is therefore a key aim of the Open Research movement, yet mounting evidence demonstrates that the practices of Open Research are implemented in ways that undermine this. In response, we convened a diverse community of researchers, research managers and funders to co-create actionable recommendations for supporting the equitable implementation of Open Research. Using a co-creative modified Delphi method, we generated consensus-driven recommendations that address three key problem areas: the resource-intensive nature of Open Research, the high cost of article processing charges, and obstructive reward and recognition practices at funders and research institutions that undermine the implementation of Open Research. In this paper, we provide an overview of these issues, a detailed description of the co-creative process, and present the recommendations and the debates that surrounded them. We discuss these recommendations in relation to other recently published ones and conclude that implementing ours requires ‘global thinking’ to ensure that a systemic and inclusive approach to change is taken.

## 1. Introduction

Open Research^1^ aims to remove barriers to accessibility and the re-use of scientific outputs by making the research process and the scholarship it yields more transparent, reproducible, accessible, shared and collaborative. Advocates suggest that Open Research will foster better and more efficient research, economic growth, increased transparency and the democratization of knowledge production [1], increase the diversity of those represented by and within research, and support the use of research outputs by policy-makers, industry, other societal actors and the public (e.g. [2]). Ensuring and increasing equity is at the heart of several of these aspects and has been championed in notable texts that sought to establish the vision for and mission of Open Research. For example, the 2002 Budapest Open Access Initiative claimed that Open Access could democratize learning and ‘lay the foundation for uniting humanity in a common intellectual conversation and quest for knowledge’ [3]. A stakeholder-driven study published in 2018 found that ‘increased equity’ was considered a ‘key success factor’ for Open Research [4], while a study that examined Open Research initiatives concluded that ‘Open science principles of openness and transparency provide opportunities to advance diversity, justice and sustainability by promoting diverse, just and sustainable outcomes' [5].

Yet, participation in Open Research is not occurring on an even playing field. The structural inequalities that shape our societies also manifest within academia, particularly in terms of unequal access to resources, and therefore some operate within the Open Research space at a distinct advantage relative to others. Therefore, simply making research ‘open’ will not automatically result in equitable outcomes. Achieving equity through Open Research requires institutional support to create the capacity to participate equally in it. By capacity, we mean the knowledge, skills, financial resources, technological readiness and motivation required to participate. Implementing Open Research without supporting capacity for less-resourced actors therefore fosters ‘cumulative advantage’ for already privileged actors (the so-called ‘Matthew effect’) and sets all others at a disadvantage [6].

In light of these circumstances, our project, ON-MERRIT (Observing and Negating Matthew Effects in Responsible Research & Innovation Transition), funded by Horizon 2020 and concluded in March 2022, investigated whether Open Research policies and practices, despite the best intentions of those implementing them, might actually foster cumulative advantage for some and disadvantage for others. We used qualitative and computational methods to examine this question across multiple stakeholder categories (including those at the periphery) and within multiple dimensions of Open Research, including its relationships with industry and policy. A key early output was our scoping review of previous literature, ‘Dynamics of cumulative advantage and threats to equity in open science: a scoping review’, published in this journal, which identified multiple ways in which cumulative advantage and disadvantage may result from current implementations of Open Research practices [6]. Our own research results^2^ further illuminate and examine such threats. From these findings, the ON-MERRIT consortium identified four key areas where threats to equity are pressing: (1) the resource-intensive nature of Open Research; (2) article processing charges (APCs) and the stratification of Open Access (OA) publishing; (3) obstructive reward and recognition practices within research and funding institutions; and (4) barriers to societal inclusion in research.

In response to these areas of concern, we used a modified Delphi method to co-create recommendations for research funders, research institutions and researchers, in partnership with a co-creation community of experts drawn from these three stakeholder groups (see appendix A for the list of named members). This process was carried out over the course of four months and involved several rounds of brainstorming, anonymized online survey, presentation, discussion and debate, and revisions with this co-creation community. At the conclusion of this process, we published a set of 30 recommendations, presented in a briefing aimed at stakeholders and titled ‘Global thinking: ON-MERRIT recommendations for maximizing equity in open and responsible research’ [7].

This paper complements and extends that briefing by: (1) situating the defined problem areas within the state of the art; (2) explicating in depth and detail our newly developed co-creative modified Delphi (CCMD) method; (3) adding depth to our recommendations by illuminating the key points of debate that arose throughout the CDMD process; (4) drawing out the underlying issues that run through the recommendations; and (5) setting our recommendations in conversation with other globally oriented and recently published sets of recommendations that serve to strengthen and mainstream Open Research practices. In what follows, we focus on concerns surrounding resources, OA publishing, and reform of reward and recognition practices. These issues relate to Open Research specifically, whereas the fourth theme presented in our policy brief, societal inclusion in policy-relevant research, is arguably applicable more to responsible research and innovation (RRI) and hence the recommendations on this topic are presented in a separate publication dedicated to that theme.

## 2. Barriers to the equitable implementation of Open Research

### 2.1. The resource-intensive nature of Open Research

Open Research practices are resource-intensive. Their adoption is based on considerable investment in terms of training, research support, and infrastructure to enable data sharing and publishing OA. Costs associated with these investments mean that those privileged in terms of institutional resources are primed to benefit most, at least initially. Indeed, better-resourced academics can be expected to be early adopters of open methods [8] for two reasons: (1) well-resourced institutions can more easily integrate open practices into their research workflows and (2) well-resourced, high-status actors tend to be early adopters in general [9]. In this, the positive effects of Open Research are offset by existing inequalities to a considerable degree, with notable effects on adoption potentials. Resource-intensity further entails that existing inequalities between institutions, nations and world regions are likely creating structural advantages and disadvantages in terms of the ability to engage in Open Research practices (also known as cumulative advantage; see DiPrete & Eirich [10]).

Proponents of Open Research practices often point to training and institutional support as a means of facilitating their adoption [2,11], yet inconsistencies among these are present even in well-resourced regions [12,13] and disparities surrounding the provision of these are greater in ‘resource-poor’ settings [14]. A European Commission survey of researchers found that most were unaware of training or support measures for Open Research, which correlates with low awareness levels among researchers of international Open Research initiatives [15]. Another, conducted by the Open Research group of the Max Planck PhDnet, found low implementation of Open Research at the institutions of those surveyed despite also finding that respondents were aware of and knowledgeable about Open Research practices and interested in implementing them [16]. Both studies point to the need for training in order to support implementation.

Where some are well resourced in this regard, the disparity in resources to support the implementation of Open Research practices could advantage already privileged actors and disadvantage under-resourced ones in a variety of ways. Those without the resources to implement Open Research practices may have their research viewed less favourably than those who do implement them, given that quality is increasingly equated with transparency [17]. Lack of resources, along with the digital divide and differences in digital literacy have been found to influence the adoption of Open Research practices [18–20], in particular the ability to implement and benefit from open data practices [21–27]. In an environment of ‘data inequalities’ [28], those at well-resourced institutions benefit while those at less- and under-resourced institutions are at a distinct disadvantage [29–31]. Therefore, the literature suggests that well-resourced actors are better equipped to implement and better able to benefit from the implementation of Open Research practices.

### 2.2. Article processing charges and the stratification of publishing

There are various routes to achieving OA to research publications. In ‘gold OA’, articles are published OA. Among the models that could be used to financially support OA publication, APCs, fees paid by authors to publish in OA peer-reviewed journals, are an increasingly common mode of support. Given that OA publishing is now a requirement or something strongly encouraged by many research institutions and research funders, they typically pay the APCs for scholarship produced by their affiliated researchers.^3^ In a world in which all researchers were equally supported by equally resourced institutions and funding, this would be an even and equitable playing field. However, given that resource stratification of research institutions exists, that research funding is limited, that the size of a journal's APC tends to correlate with its prestige [32,33], and that OA publishing may give authors a citation advantage [34,35], the APC model appears to create inequity in the OA publishing space, with well-resourced researchers potentially at an advantage relative to those who are under-resourced [8,32,33,36–44]. In particular, research has shown that women [45], scholars within the social sciences and humanities [46], those from middle- and lower-income nations [47,48] and early career researchers [49] experience disadvantage relative to others when it comes to OA publishing. Since support for APCs is typically available from funders or research institutions, actors beyond the academy, such as civil society organizations or industry, are also excluded from OA publishing [50,51]. Additionally, those from the periphery are more at risk of exploitation by low-quality, predatory journals that have colonized OA publishing in certain geographies, which leads to their contributions being dismissed and overlooked [52,53]. All of which suggests that OA publishing, as it currently operates, is fostering cumulative advantage and disadvantage.

### 2.3. Obstructive reward and recognition practices

There is a growing body of research that shows that institutional norms and practices of reward and recognition do not support (enough) the values of Open Research and the uptake of its practices. Further, existing norms and practices often undermine these or get in the way of those who wish to practice Open Research. Research indicates that criteria that relate to or support Open Research are rare across institutional policies at research institutions around the world [45,54–56]. Simultaneously research shows that quantitative measures dominate in promotion and tenure processes at EU institutions, with number of publications being the most common measure used [57]. This finding is consistent with other research that found that reward and recognition practices give too much weight to quantitative measures over qualitative ones [58,59]. Troublingly, other research has found that, beyond an overreliance on them, quantitative measures including the journal impact factor (JIF), among others, are being misused in reward and recognition practices [45,55,60–65]. Meanwhile, efforts to support and foster Open Research infrastructures, like the often unpaid work by those who create open source code [66], are rarely valued in reward and recognition processes [65]. Other research has demonstrated an overall lack of institutional support for Open Research [13], which is reflected in the fact that researchers believe that Open Research and other qualitative aspects of their work are not valued by their peers [45], nor by those carrying out reward and recognition processes [67]. Therefore, though many researchers embrace Open Research values and support its practices in principle [16,68], many do not implement them out of fear that doing so may harm their careers [60,67,69–71] and because they lack institutional incentives and support to do so [13,57]. Cumulatively, these institutional practices and the cultures they foster contribute to producing what is known as the ‘attitude–behaviour gap’ with regard to the uptake of Open Research practices [72]. The clear take-away from this tranche of the literature is that Open Research is neither supported nor rewarded effectively at (most) research institutions (nor by academic culture more broadly).

### 2.4. Equity implications of these issues

The centrality of institutional resources to the practice of Open Research should be of great concern to its advocates for several reasons. Primary among them is the inequity that is fostered by this unequal playing field when Open Research practices are increasingly prioritized by science policy-makers and funders. As a consequence, well-resourced actors who are supported to implement a diverse range of Open Research practices will benefit from policies and funding opportunities that privilege them while less-resourced actors will be disadvantaged in such a field of competition. This reality could undermine the ability of less-resourced actors to collaborate with well-resourced ones, further marginalizing them from funding opportunities and accrued reputation and success on the global stage. The literature reviewed therefore implies that the expectation of Open Research practices without the equitable distribution of resources and support for implementation creates a cumulative advantage for already privileged actors and disadvantage for all others.

Within this context, the disparities caused by the current system of OA publishing are especially troubling in their implications. Within policy-making, there is an increasing interest in relying on research outputs to shape responses to problems and an emphasis on the potential utility of Open Research to foster this [73]. The inequitable trends within OA publishing could mean that only the scholarship and viewpoints of the most privileged scientific actors have a seat at the table when work on global societal challenges is done. How these challenges are framed and understood, the questions that are asked about them, how research is conducted in response to them, and the solutions that are put forward will remain exclusionary processes if these trends continue. This is not only unjust and inequitable, but also not the best science, given that the contributions of the majority are left out. This reality is in stark contrast to the aims of Open Research.

To counteract these trends, in support of the equitable uptake and conduct of Open Research, a shift in the culture and norms of reward and recognition practices among research institutions and funders is needed. For Open Research to flourish in an equitable manner, credit and recognition must be given for implementing Open Research practices and creating Open Research resources, which necessarily implies a shift toward qualitative measures and away from quantitative ones. Efforts to achieve this shift are already underway, most notably those related to the Declaration on Research Assessment (DORA)^4^ and the Leiden Manifesto for Research Metrics [74], which together recommend shifting away from quantitative metrics in favour of the qualitative evaluation of research contributions, and institutional flexibility that recognizes the diversity of disciplinary approaches to research and scholarship. Speaking more broadly to the support of Open Research (including reward and recognition processes), the UNESCO Recommendation on Open Science [75] takes a global, policy-oriented view to supporting the implementation and growth of Open Research practices. The ‘key objectives and areas of action’ of the UNESCO recommendation reflect the problem areas as we have described them here and therefore our recommendations are in alignment with these. Our contribution differs in its focus on issues of equity within Open Research, and its focus on the role of European institutions and actors in fostering equitable Open Research (though like UNESCO, we maintain a global view of the issues). Having a meso- and micro-orientation, our recommendations provide more specific, tangible and immediately actionable suggestions for institutions and actors within the European research landscape.

## 3. Co-creation methods

### 3.1. A co-creative modified Delphi design

We used a modified Delphi method to co-create recommendations with a group of stakeholders representing researchers, research-performing organizations and funders. The traditional Delphi method is iterative, anonymous and survey-based, and typically involves multiple cycles of gathering expert views, aggregating and synthesizing them, presenting the outcomes to the experts and then beginning the cycle again [76,77]. The use of ‘modified’ or ‘hybrid’ Delphi methods is common in research that seeks to generate consensus around responses to a problem that result in recommendations to institutional leaders or policy-makers [76,78,79]. These approaches use a combination of established qualitative social science methods, like focus groups and workshops, to craft a modified approach that includes traditional ‘closed’ anonymous surveys with ‘open’ face-to-face discussion [78,79]. Modifying the Delphi method in this way allows it to be used as a co-creation method—now commonly deployed as such in Open Research and RRI projects [80–84].

Situated within RRI, which centres co-creation of research agenda and contents to ensure that research and innovation are responsive to the needs and values of society and produce societally good outcomes [85–89], our CCMD approach was fully co-creative and reflected now established approaches, beginning with an open-ended survey to gather recommendations from our multi-stakeholder group (similar to the approaches taken in [90–93]), and progressing as a continually co-created, iterative, consensus-driven process until the recommendations were finalized over the course of eight phases, organized as follows:

**Table:** 

Phase 1	Anonymous survey to gather recommendations in response to four problem areas
Phase 2	Workshops with each stakeholder group to discuss proposed recommendations and gather more
Phase 3	Compilation and synthesis of proposed recommendations and revisions based on cumulative feedback
Phase 4	Anonymous survey to establish consensus and gather additional feedback on revised recommendations
Phase 5	Sorting of recommendations as at consensus or not and proposed revisions based on received feedback
Phase 6	Final workshop to discuss and collaboratively revise recommendations not yet at consensus
Phase 7	Collaborative validation and minor revisions to list of recommendations
Phase 8	Final validation, minor revisions and publication of recommendations policy brief

**Figure 1.**
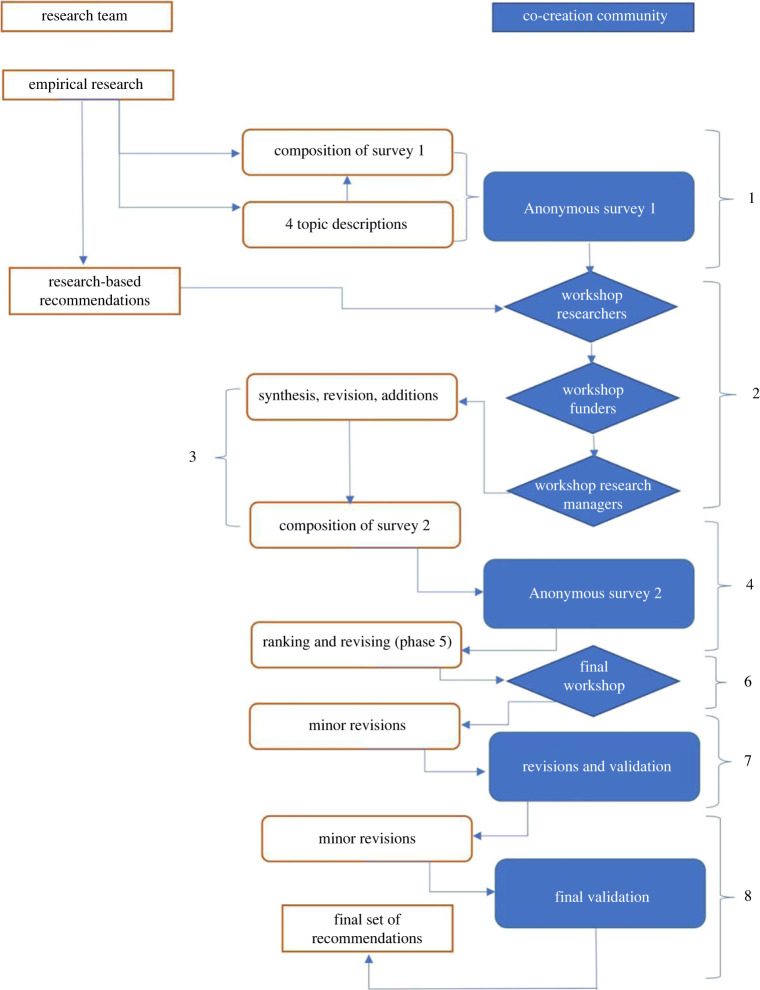
Co-creative modified Delphi process with Phases 1–8 noted.

In what follows, we describe the components and process of our CCMD method and describe each phase in detail, as illustrated in figure 1.

## 4. Preliminary work

### 4.1. Problem areas, topic descriptions and internally created recommendations

The synthesized results of the ON-MERRIT project yielded four key areas of concern where current practices for implementing Open Research could be exacerbating existing forms of cumulative advantage or creating new ones: (1) the resource-intensity of Open Research, (2) APCs and the stratification of OA publishing, (3) societal inclusion in research and policy-making, and (4) reform of reward and recognition practices to foster the expansion of Open Research. Having identified these four areas, we crafted a brief topic description for each that would serve to inform the process of co-creating recommendations. Simultaneously, we internally co-created some recommendations with our project consortium members, which were included for discussion in the series of Phase 2 workshops, alongside those offered by the external co-creation community.

### 4.2. Co-creation community recruitment and participation

We used purposive sampling to gather our co-creation community from our network of colleagues across Europe working at research funders and research-performing organizations to gather three distinct groups composed of researchers, funders and research managers. We aimed to recruit a diverse sample, in terms of gender, ethnicity, career stage and institution within each stakeholder group. Our sample consisted of 10 participants in the researcher group, 9 in the funder group, and 10 in the research manager group, for a total of 29 participants (12 men and 17 women, ranging from early career to more senior). Together, they represent 27 organizations across 16 countries, primarily located in Europe, and with one participant each from Africa and Latin America. The list of named members is presented as appendix A.^5^

Participation by our co-creation community was inconsistent and waned across the full CCMD process (table 1). While all participated in the first series of workshops, only 65.5% of them completed the Phase 1 survey prior to these events. Just under half of them participated in the Phase 4 survey (despite email reminders), and just under a third participated in the Phase 6 final workshop. Participation dropped further during Phases 7 and 8. Despite the drop in participation, we maintained diversity across the participant categories throughout the process. However, throughout the process research managers, representing research institutions, were the most engaged group.

**Table 1.** Participation across the CCMD process.

	Phase 1	Phase 2	Phase 4	Phase 6	Phase 7	Phase 8
researchers	4	10		2	1	1
funders	5	9		3	2	1
research managers	10	10		4	3	3
total	19	29	14	9	6	5

## 5. Description of the full CCMD process

### 5.1. Phases 1, 2 and 3: initial survey, workshops and synthesis

During **Phase 1**, we used a set of three identical anonymous surveys (one for each stakeholder group) to gather recommendations in response to the four topic areas from our co-creation community, asking them to specify whether their recommendations were general in nature or targeted to funders, research-performing organizations and/or researchers, and compiled internal recommendations from the ON-MERRIT project consortium. We received a total of 19 responses to this survey: 4 from researchers, 5 from funders and 10 from research managers. Researchers contributed 9 recommendations, funders contributed 39 (some duplicate but aimed at different target audiences), and research managers contributed 139 (also numerous duplicates aimed at different audiences).

**Phase 2** consisted of three online 3-hour workshops, one with each stakeholder group. These workshops were organized as four separate discussions of the four defined problem areas and the recommendations so far compiled in response to each. These workshops were recorded, annotated and transcribed, and the meeting chats saved to facilitate Phase 3. We prompted discussion of the recommendations by asking participants to identify and comment on those recommendations that made a strong impression upon them, whether positive or negative, and invited them to offer additional recommendations. This approach yielded active discussion and debate that made it clear which recommendations were broadly supported, which were not, and which required revision.

We took a cumulative approach to compiling recommendations, during this phase, adding each group's survey responses to the existing set of recommendations from the prior workshop as the phase progressed (table 2). During the first workshop held with researchers, we presented and discussed a total of 49 recommendations, 40 of which were generated internally. The results of the survey for the second group of workshop participants, research funders, were synthesized with the existing set of co-created recommendations from workshop one, which resulted in an updated set of 79 co-created recommendations presented in workshop two. The same process was carried out for workshop three with research managers, during which 93 recommendations were presented and discussed. Therefore, cumulatively Phases 1 and 2 resulted in a progressively revised and expanded set of co-created recommendations that were markedly different from the first set presented in the first workshop. (Bear in mind that the number is high because some recommendations were presented multiple times but aimed at different target audiences.)^6^

**Table 2.** Co-creation of recommendations across Phases 1 and 2.

topic	internal	researcher survey	researcher WS	funder survey	funder WS	RM survey	RM WS
APC	6	6	12	12	22	48	24
inclusion	18	0	18	12	23	31	27
reform	12	1	13	7	22	23	24
total	40	9	49	39	79	139	93

During **Phase 3**, we compiled all feedback gathered during Phase 2 in order to revise, synthesize and hone the list of co-created recommendations, drawing on transcripts, notes and meeting chats to do so. This process included flagging any newly offered recommendations for internal discussion. We evaluated the 93 recommendations generated during Phase 2 and determined, based on the consensus that emerged, whether to reject, keep as is, or revise each one. We defined consistent negative feedback to a recommendation as consensus for rejecting it and not carrying it forward and otherwise sought to revise recommendations based on consistent and recurring feedback. While revising, we combined any recommendations that were close in wording, intended meaning, or spoke differently to the same issue or set of linked issues. Cumulatively, we revised 13 recommendations, revised and consolidated 58 down to 20, struck 16, and reassigned 6 to a different category (2 of which were classed as general recommendations which were taken out of the process and set aside, and 4 of which were then combined with already existing recommendations in these other categories). This resulted in 33 revised recommendations at the end of Phase 3.

### 5.2. Phases 4, 5 and 6: second survey, final workshop and synthesis

During **Phase 4**, we presented the revised set of co-created recommendations via an anonymous survey to our co-creation community. Participants were asked to review each recommendation and consider whether they agreed with the recommendation as written. Response was mandatory and options included, ‘Yes, I agree’, ‘Yes, but with minor revisions', ‘Yes, but with major revisions’ or ‘No, I disagree’. When respondents selected an answer that indicated revision or disagreement, a mandatory text box appeared and prompted them to provide their suggested revision to or reason(s) for rejecting the recommendation. This survey yielded 4 partial and 14 full responses from our 29 co-creation community members (survey participation was not disaggregated by stakeholder category during this phase).

We used a consensus scale to organize the survey results, ranging from *at consensus* (recommendations to which participants agreed as stated, or agreed with minor changes), *nearly at consensus* (those that received no more than one suggestion for major changes and no disagreements), *further from consensus* (those that received no more than one disagreement or two or more suggestions for major changes) and *far from consensus* (those that received two or more disagreements and/or three or more suggestions for major changes). Based on this scale, 21 of the 33 recommendations presented were already at consensus, 4 were nearly at consensus, 6 were further from consensus and 2 were far from consensus. Therefore, a total of 12 were not yet at consensus, and to these respondents provided qualitative suggestions for revisions or reasons for rejecting.

Taking on board the qualitative feedback received through the Phase 4 survey, during **Phase 5** we revised recommendations to the best of our ability—given that there were some conflicting and debatable points of feedback that necessitated further discussion with the participants—and proposed to strike some. In keeping with the survey results, we retained 10 recommendations as they had been stated in the survey and we made minor revisions to 15 of them, including some already considered to be at consensus (making only minor revisions to improve the writing and readability or to clarify the applicability). We made more substantial revisions to 5 and major revisions to just 1, and recommended striking 2 that received overwhelmingly negative feedback from survey respondents.

**Phase 6** consisted of the final co-creation workshop, to which all participants in our co-creation community were invited. During this 2-hour online workshop, we facilitated discussion and real-time collaborative revision of the 12 recommendations that were not yet at consensus, including those that were recommended to strike, until all participants were satisfied with the revisions or the decision to reject. We considered consensus to be a full group agreement. Nine members of our co-creation community attended this workshop, including 4 research managers, 3 funders and 2 researchers. We ultimately reached a consensus wording for 10 recommendations and reached consensus about eliminating the 2 recommendations that we had proposed to strike. At the end of this co-creation workshop, we had a consensus-based list of 31 recommendations with which to move forward. These included 5 in the APC category, 10 each in Inclusion and Reform and 6 in the Resources category.

### 5.3. Phases 7 and 8: final revisions and validation

During **Phase 7**, we made additional minor revisions for writing and readability and collectively decided to strike one more recommendation that we deemed to be out of scope for funders, research institutions and researchers. This reduced our list of recommendations from 31 to 30, which were shared with our co-creation community via a Google Doc for any final suggested revisions. Six members of our co-creation community participated in this process, including 1 researcher, 2 funders and 3 research managers.

During **Phase 8**, we made final revisions based on the feedback received during Phase 7 and shared the recommendations one final time with those members of the co-creation community who participated in Phase 7. Five of them participated, including 1 researcher, 1 funder and 3 research managers. After a few final revisions were made based on their feedback, we finalized and published 30 co-created recommendations as a digital policy brief [7].

## 6. Results

In this section, we present the qualitative results of our Delphi process by topic area, beginning with the recommendations themselves, and including important debates surrounding them and key decisions made by the co-creation community throughout the process. Our findings demonstrate that overall, the APC topic generated the most discussion and debate. It generated the most recommendations from each of our stakeholder groups during the Phase 1 survey, signalling its universal importance. Recommendations offered in this category also proved to be the most controversial, generating the most debate throughout the process and requiring more phases than other categories to reach consensus. While debates arose around recommendations in the other categories as well, consensus on most recommendations within Resources and Inclusion were reached by Phase 4. In what follows we first present the final co-created recommendations for each topic area (excepting Inclusion) before offering more details of the CCMD process for each. We interpret and discuss the implications of these findings in the subsequent discussion section of this paper.

### 6.1. Resource-intensity of Open Research

(1) Funders, institutions and researchers should encourage and support the use of sustainable, shared Open Research tools, training materials, and infrastructure, to foster inclusivity, reduce costs and promote open standards.
(2) Funders, institutions and researchers should strategically prioritize collaboration and partnership with less-resourced regions/institutions, to build knowledge of and capacity for Open Research via direct exchange of knowledge and resources amongst actors and communities of practice.
(3) Funders and institutions should require Open Research and RRI practices wherever appropriate and support the associated costs.
(4) Funders and institutions should provide basic and advanced training courses on Open Research and RRI tailored to specific contexts (including disciplines, career stage and specific research areas), investing in more trainers to directly support researchers and making training materials open to anyone who may wish to use them.
(5) Funders and institutions should encourage and support maximal transparency regarding the costs associated with Open Research practices. Additionally, they should support research to understand the costs associated with not doing Open Research to create a baseline for understanding the economic impacts of Open Research.
(6) Funders and institutions should commit resources to (meta-)research to investigate and monitor equitable uptake of Open Research and RRI globally, and expand or develop (open) infrastructures to sustainably enable this meta-research.

As was mentioned in the introduction to this section, we found that overall, consensus was relatively easily reached for recommendations within the Resources category. While there was some discussion of how exactly to phrase some recommendations, there was hardly any debate about the merits of any of the proposed recommendations. During early phases of the CCMD process, some of the nuances discussed included the following:

— Care should be taken to direct some resource investment to the actors who are already engaging in innovative and open practices and are currently under-supported in this work, like communities of practice [94], civil society or non-governmental organizations and early career researchers.
— Enabling costs associated with OA and open data to be covered by project funds, regardless of the timing of the outputs.
— The importance of transparency around baseline research costs in order to accurately measure the cost/benefits of Open Research.
— The need for careful attention to how resources to support Open Research are shared between well-resourced and less-resourced institutions, particularly across global north/south and colonizer/(formerly) colonized divides, and of recommendations to take differing resource levels into account in terms of what is a reasonable ‘ask’ for a given institution.
— The importance of mentorship, especially training *senior* researchers to improve the implementation of and guidance for Open Research practices in research and educational settings.
— The importance of all training materials and platforms being open in nature.

Results of the Phase 4 survey showed that all but one recommendation were at consensus at this stage. Our co-creation community expressed concern about the recommendation to have dedicated funding lines for Open Research, stating that it implied that funders would provide unlimited funding to publishers via APC charges; that instead, emphasis should be placed on supporting ‘diamond OA’ (as is also noted in discussion subsequent of the APC recommendations); that dedicated funding lines ‘fetishize’ Open Research when in fact it should be the default; and relatedly, that the phrasing suggested that non-open research is the norm and is legitimate, while participants frame it as illegitimate. Based on this considerable negative feedback, we struck this recommendation during Phase 5. No substantive discussions or debates occurred around this category of recommendations in subsequent phases.

### 6.2. Article processing charges and the stratification of publishing

(1) Funders, institutions and researchers should collectively demand greater transparency from publishers on publication costs, regarding prices and services, and (where possible) support open infrastructures to collect this information.
(2) Funders, institutions and researchers should support alternative publishing models where those show potential to be more inclusive, including consortial funding models for open publishing infrastructures which support OA publishing with no author-facing charges.
(3) Funders, institutions and researchers should encourage and support the use and maintenance of sustainable, shared and open source publishing infrastructure, to reduce costs and promote open standards.
(4) Institutions and researchers should ensure the accepted version (or later) of peer-reviewed works are deposited in an open repository.
(5) Funders and institutions should consider supporting authors' right to self-archive publications by implementing rights retention strategies (RRSs).

As mentioned in the introduction to this section, how to respond to APCs and the stratification of authorship in OA publishing generated the most engagement across our stakeholder groups throughout the CCMD process. In the early phases, considerably more recommendations were generated in this category than in others, and around these, more discussion and debate took place. The recommendations offered proved to be the most controversial, in the sense that a greater number were struck after the Phase 2 workshops (half of all those struck at this time), and this category had the fewest already at consensus as a result of the Phase 4 survey (fewer than a third). And at this stage, APC was the only category for which we moved to strike recommendations (two of them) based on the quantitative and qualitative feedback provided via the survey. Therefore, the APC category dominated the discussion during the final focus group conducted during Phase 5, during which considerable discussion took place to finalize the wording of those not yet at consensus, and during which consensus emerged to strike the two recommendations that we had suggested.

The topics that generated the most debate included:

— How best to recommend a route to OA publishing, given the various options available and the issue of author-facing costs associated with some models.
— How best to self-archive as an alternative to OA publishing, whether self-archived texts must be already peer-reviewed, and how best to recommend doing so, given the fear that researchers, especially those early in their careers, could face rejection from publishers for doing so.
— Similar concerns were expressed surrounding a recommendation for institutions to support the RRS^7^ for authors.
— The pros, cons and feasibility of funders installing APC caps.
— The pros and cons of APC waiver programmes (and increasing them), especially given that they ultimately support rather than challenge the APC system.
— Encouraging researchers to publish in community owned journals or other alternative publishing outlets with no or low-cost APCs, and/or to select journals based on criteria other than prestige.

Ultimately, throughout various phases, our co-creation community decided to strike proposed recommendations pertaining to APC caps, increasing waiver programmes, and encouraging researchers to publish in alternative outlets and/or to use criteria other than prestige when selecting a journal. For the latter, our co-creation community reached consensus on the position that researchers should always choose a publication outlet that best suits their research, regardless of its open/closed status and APC. A combined recommendation pertaining to self-archiving and RRS was debated and ultimately preserved, with revisions to the wording to recommend that institutions should ‘consider’ supporting these initiatives, rather than absolutely doing so.

### 6.3. Reform of reward and recognition

(1) Funders and institutions should support a change in assessment culture, moving beyond narrow quantitative indicators (e.g. of publication and funding acquisition) to value quality, openness (where appropriate), collaboration and responsibility in research, and recognize the full range of academic tasks.
(2) Funders and institutions should make reward and recognition processes flexible to respect diversity in its many forms, including disciplinary differences, national assessment frameworks, institutional values and missions, and differing experiences and career trajectories related to gender, race/ethnicity, age, etc.
(3) Funders, institutions and researchers should collaborate with all stakeholder groups at local, national and global levels to define and implement reformed reward and recognition practices.
(4) Funders and institutions should encourage and support coordination activities to foster knowledge-sharing and awareness of best practices regarding reform of research assessment practices, especially between experienced and less-experienced actors.
(5) Institutions should designate institutional leaders or teams responsible for research culture and research improvement to guide reforms.
(6) Funders and institutions should implement inclusive processes towards reaching consensus on aims and means for reforming reward and recognition processes.
(7) Funders, institutions and researchers should ensure that all relevant stakeholders, especially persons involved in hiring and assessment, are part of efforts to reform reward and recognition processes.
(8) Funders and institutions should ensure that all those involved in assessment processes are suitably trained in best practices, as collaboratively defined by all stakeholders.
(9) Researchers should, where possible, lead by example with regard to Open Research and RRI and as part of a diverse and representative coalition, push for reform within their institutions.
(10) Institutions should ensure sustainable career pathways are available for research support staff facilitating Open Research.

Participants across our three stakeholder groups engaged consistently with the topic of how to reform reward and recognition practices in order to best support the uptake of Open Research practices. We note, however, that in the initial Phase 1 survey, researchers offered just one recommendation within this topic (though they engaged actively during the Phase 2 workshop with the internally generated recommendations that we offered). This perhaps signals that researchers do not believe that they have the power to change reward and recognition practices, or perhaps that they are unsure of how to do so, given how culturally entrenched these practices are.

The recommendations offered within this category fell under four broad topics, including the importance of a collaborative approach, who should lead the way and how, the importance of grass-roots efforts, and how institutions can support the growth of Open Research practices through specific reforms. Throughout the CCMD process, the offered recommendations were mostly non-controversial. During Phase 3, just one was struck based on Phase 2 discussions and none were proposed for deletion later in the process. However, debate did ensue from Phase 2 onward about the nuances and specifics of many of them. The topics that generated the most discussion and debate included:

— The importance of recommending collaborative efforts with all stakeholder groups to define and drive reforms (at local, national and global levels), coupled with the idea that funders may need to push research institutions to implement reforms, and couched within concern that the voices and power of those privileged by the existing system could hamper reform efforts.
— A sense of caution against standardizing aspects of Open Research in policies (for implementation and monitoring) that might follow recommendations, in the interest of recognizing epistemic diversity and the diversity of institutional resources, needs and profiles. Ultimately a recommendation that pointed to standardization was struck based on consensus feedback against it.
— The potential danger for early career researchers to take leading positions on this issue and the importance of those more senior in their careers, and therefore with job security, to take the lead in driving change.
— Simultaneously recognizing that people at various career levels can act in the service of change through a diversity of means.
— The importance of all stakeholders involved in hiring and the reward and recognition process being part of efforts to reform (as collaborative stakeholders and in terms of training in new practices).

Ultimately, the discussions within this topic area were mostly focused on nuances of how to recommend something rather than debates of whether or not to recommend something, indicating that a certain level of consensus on how to approach this issue already exists among interested stakeholders.

## 7. Summary of results

In sum, we found that the topic of OA publishing and the problem of APCs generated the most attention, engagement and debate throughout our CCMD process. Meanwhile, the topics of the resource-intensity of Open Research and the need to reform reward and recognition to support the uptake of Open Research were, on the whole, non-controversial and consensus was quickly and easily achieved on which recommendations to make in response to these issues.

## 8. Discussion

The recommendations that we generated with our co-creation community are meant to respond to critical challenges within the Open Research landscape: the uneven distribution of resources across institutions and geographies; the impact this has in particular on the ability of researchers to publish in high-author-facing-cost journals; and the lack of institutional support, in the form of reward and recognition, for Open Research practices. At issue here is not simply these challenges themselves, but that some are able to succeed and thrive in such an Open Research landscape, while others are left behind, due largely to unequal access to resources. Therefore, these recommendations are meant to correct inequalities and injustices in this landscape and to ensure that the further uptake and diffusion of Open Research practices (and cultures and infrastructures) proceed with equity at the forefront of institutional policies and practices.

Taking a broader view, a key underlying theme emerges through all these recommendations and across the individual issues: *the reflexive need to embrace the values of Open Research in order to fully implement and realize these recommendations* so that changes carried out are themselves collaborative and transparent in nature and the resources that support them are open and freely available to all. Most of our recommendations include prescriptions for transparency of processes, flexibility and diversity of policies and practices with an eye to epistemic diversity, open resources and infrastructures and a socially open approach, in terms of collaborating and sharing resources in pursuit of solutions. This suggests that our co-creation community believes that Open Research must be achieved not just through reform of the research process itself, and how it is administered, but also through how we behave as actors within the system of research and knowledge production. In other words, our results seem to tell us that openness begets openness; that fostering the diffusion of Open Research practices requires a socio-technical shift in the norms, values, practices and rituals that constitute research, as well as in the infrastructure, processes and (physical and digital) artefacts that enable it, structure it, produce it and are produced by it.^8^ And, the aims of these shifts and how they are implemented must be collaboratively defined, carried out and the benefits of them shared.

**Figure 2.**
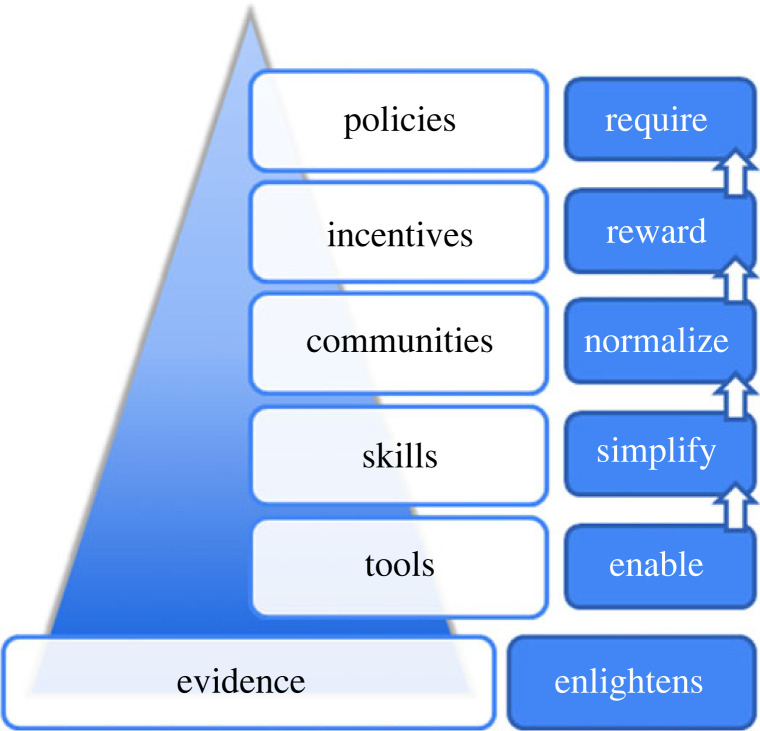
An integrated, evidence-based approach to fostering the growth and spread of Open Research, adapted from Nosek [96]*.*

We refer to such an approach as ‘global thinking’, a term that has two distinct senses [95]. Firstly, thinking should be ‘global’ in that approaches should be joined-up to target reform of the research ecosystem as a whole rather than taking atomistic policy actions that target specific aspects of Open Research while leaving others unaddressed. This also means coordinating action across the stack of elements identified by Nosek's strategy for culture change [96] (figure 2): creating tools (infrastructures and services) to make it possible, equipping researchers and others with skills to make it easy, networking communities to make Open Research practices the norm, revising incentives to make it rewarding, and implementing policies to encourage or demand (where appropriate) Open Research practices. All this should be further supported by greater support for meta-research to provide the effective evidence base from which to judge the efficacy and effects of these actions. Much work is already in place across all these elements. Linking and building on such initiatives is an essential task.

Specific to our recommendations, and referencing figure 2, we highlight the following.

**Tools** should be open and shared in an effort to help lower the cost of entry into Open Research and to more equitably distribute resources among those who already have (a lot of) them and those who do not. This is particularly important across the global north/south divide and can be fostered in part by equitable research collaborations and partnerships. Doing so will spread resources more broadly and help to make the uptake of Open Research practices more efficient by avoiding the ‘reinvention of the wheel’ that results from siloed thinking and action.

The development of **skills** must be supported in ways that respect epistemic diversity as well as institutional diversity. Not all Open Research practices are appropriate for all types of research (and scholarship), and at the same time, training for Open Research practices must be flexible so as to account for the variation of practices across research areas. Similarly to the approach taken with tools, materials and courses that support the development of skills should be open and shared for the same reasons.

**Communities**, broadly conceived and at a multitude of levels, play an important role in culture change by giving space for dialogue, collaboration, creativity of solutions, and the creation of new norms that reinforce, foster and celebrate Open Research. They are also important venues for discussing the problems that exist in order to illuminate them more broadly, for fostering solidarity around them, and for generating momentum for action.

**Incentives** to support Open Research, particularly in the form of reward and recognition practices, are critical to its spread. A need for a culture change to support the values of collaboration and openness that underpin Open Research is clear, and should shift the focus from assessing quantifiable research outputs to qualitative research behaviours. The revision of existing incentives and implementation of new ones must themselves, like other aspects discussed above, be collaborative, consensus-based, transparent and open, as well as flexible and responsive to epistemic diversity. In addition, the critical research support roles that allow for the implementation and operation of Open Research practices must themselves be incentivized through long-term (rather than project-based) and appropriately compensated employment contracts.

Finally, **policies**, like incentives, should be collaboratively and transparently defined by all stakeholders, and also flexible and responsive to epistemic diversity. Context matters greatly in this regard, in the sense that policies will necessarily differ across contexts where Open Research practices are already present and strongly supported with a multitude of resources and incentives versus those where virtually no support yet exists. And importantly, policies that require the tracking and monitoring of all aspects of this step-wise process should be implemented, and these too should be transparent and their outcomes openly shared.

The second sense of global thinking that we wish to elaborate is that the problem areas targeted by our recommendations desperately need greater shared understanding and dialogue amongst stakeholders from across the world in order to set mutual expectations and responsibilities. In particular to avoid policy priorities being set by Global North actors without due reflection on potential (unintended) ill consequences, especially for historically disadvantaged groups. To avoid this, participation in global initiatives to create shared visions for Open Research should be a baseline activity of all who wish to implement our recommendations. In encouraging ‘global thinking’, however, we explicitly do not wish to imply the desirability or possibility of achieving a ‘one-world’ vision for all of Open Research. As the OCSDnet Manifesto's emphasis on ‘situated openness’ to address the ‘ways in which context, power and inequality condition scientific research’ reminds us, openness is not an absolute and its optimal levels across epistemic processes may differ greatly in different contexts.^9^ Respecting those contexts, and the autonomy of distinct groups to govern their own knowledge processes, remains crucial. Yet, inviting common conversation is crucial precisely to safeguard this autonomy. This should not only occur between regions, but since change happens at local, regional, national and global scales, collaboration across these scales is also critical.

In calling for global thinking, we are not suggesting that giant strides are not already being made in this direction. The OCSDnet Manifesto, mentioned above, is a key touchstone. In addition, the recent initiative to create a shared global vision such as the UNESCO Recommendation on Open Science [75], adopted in November 2021, shines a light. Its ‘key objectives and areas of action’ include promotion of a common understanding of Open Research; ‘developing an enabling policy-environment’; investment in infrastructures, services and human resources in support of Open Research; ‘fostering a culture of open science and aligning incentives' for it; promoting various approaches to Open Research across the scientific process; and ‘promoting international and multi-stakeholder cooperation’ to help reduce ‘digital, technological and knowledge gaps'. Aligning strongly with our own set of recommendations, this document speaks specifically to governments of UNESCO's 193 member states, which offers hope and promise for action at both global and national (government) levels that would support the implementation of recommendations like ours at the institutional level. Published simultaneously, the International Science Council's position paper titled ‘Science as a global public good’ emphasizes the importance of Open Research values and practices of transparency and openness (in terms of access) to ensure that research outputs serve the public [97]. These, and other global initiatives like DORA and Plan S, signal a positive direction of travel in terms of support for Open Research.

## 9. Conclusion

As our research and that of others highlighted in this paper has demonstrated, current implementation practices of Open Research are producing cumulative advantage for the few and disadvantage for the many. Therefore, we submit that our co-created recommendations offer a unique perspective, with equity at the centre, and that as such they are an important complement to other contributions in this vein.

While we believe in their unique value, we also admit their limitations. Firstly, these recommendations are targeted at funders, research institutions and researchers as the three groups most closely affected by these issues and best positioned to take action in response to them. However, change requires action from a variety of actors, including governments, civil society actors, industry and publishers. We recognize this and also recognize that some of the recommendations that follow implicate action among these other actors. In particular, governments have a crucial role in ensuring this agenda is supported through their policies which should seek to maximize academic and scientific understanding amongst their populations, while publishers of scholarly work should also act in responsible ways oriented to equity and not merely profits.

Secondly, while our recommendations have been written via a co-creative process with global actors, and with global implications in mind, we nonetheless concede our collective standpoint may influence our positions. And, as noted in our methods section, we experienced a drop in participants throughout the CCMD process, meaning that the fine tuning of the recommendations in the latter phases was carried out by a small group of highly engaged stakeholders. This too may have influenced our outcomes. The recommendations are, however, written in a way that allows for broad applicability, leaving it up to the relevant actors to determine how to implement them. And in terms of tone and (some) content, they align with the UNESCO recommendations and others, which illustrates alignment with the views of a much broader set of stakeholders.

We conclude by emphasizing that the ways in which we implement change today will have long-lasting consequences for the kind of Open Research ecosystem we inhabit tomorrow. For that future to be one more equitable than our present, critical consideration must be given to the ways in which agendas of openness are shaped by those in positions of power and privilege, and might hence reflect or even reinforce global dynamics of inequality. Given its commonly held aim of increasing equity, any potential for Open Research to actually drive inequalities must be taken seriously by the research community in order to realize the aim of making research truly open and collaborative, and those who practice it free from bias in doing so and equally open to the system's rewards and recognition.

## Data Availability

The primary data are qualitative and are confidential in order to preserve the privacy of those who participated in generating them.

## Footnotes

1 We use the term Open Research rather than Open Science to be inclusive of research that is not construed as science, strictly speaking.

2 See here: https://zenodo.org/communities/on-merrit/?page=1&size=20.

3 To date ROARMAP, the Registry of Open Access Repositories Mandatory Archiving Policies, has compiled Open Access policies from more than 900 organizations around the world, composed primarily of research institutions but also research funders. See here: https://roarmap.eprints.org/.

4 https://sfdora.org/.

5 Most participants agreed to be named publicly but a few chose to go unnamed.

6 During Phases 1 and 2, we did not apply consensus rules. Rather, we treated these as brainstorming phases and allowed all ideas to be contributed through the open-ended survey and series of workshops.

7 https://www.coalition-s.org/rights-retention-strategy/.

8 See Geels (2004) for a definition of socio-technical systems: https://www.sciencedirect.com/science/article/pii/S0048733304000496.

9 https://ocsdnet.org/manifesto/open-science-manifesto/.

## Appendix A. Co-creation community

These recommendations were drafted via a co-creation process with members of the stakeholder groups to which they are addressed (funders, research institutions and researchers). We gratefully acknowledge the contributions of all these community members, including those listed below, as well as those who advised they did not wish their name to be shared.

We especially thank Juan Alperin, Neil Jacobs, Valerie McCutcheon and Jorge Noro for very constructive comments and suggestions on advanced drafts of our Recommendations Briefing.

**Table:**

Alperin, Juan Pablo	Simon Fraser University, Canada
Berezko, Oleksandr	Lviv Polytechnic, Ukraine
Gadd, Elizabeth	University of Glasgow, Scotland
Grandits, Kristina	Austrian Research Promotion Agency (FFG), Austria
Griessler, Erich	Institute for Advanced Studies, Austria
Himanen, Laura	Tampere University, Finland
Hope, Hannah	Wellcome Trust, England
Jacobs, Neil	UK Research and Innovation, England
Karner, Sandra	The Interdisciplinary Research Centre for Technology, Work and Culture (IFZ), Austria
Kotar, Mojca	University of Ljubljana, Slovenia
Klarenbach, Magdalena	Fundacja Otwarty Plan, Poland
Labastida, Ignasi	University of Barcelona, Spain
Macgregor, George	University of Strathclyde, Scotland
Masuzzo, Paola	Institute for Globally Distributed Open Research and Education, Belgium
McCutcheon, Valerie	University of Glasgow, Scotland
Mendes Moreira, João	Foundation for Science & Technology, Portugal
Meyer, Dagmar	European Research Council Executive Agency, Belgium
Noro, Jorge	University of Coimbra, Portugal
Osano, Philip	Stockholm Environment Institute, Africa Centre, Kenya
Pais, Clarisse	Instituto Politécnico de Bragança, Portugal
Pelagotti, Anna	European Research Council Executive Agency, Belgium
Rollo, Maria Fernanda	Universidade Nova de Lisboa, Portugal
Rovelli, Laura	Latin American Council of Social Sciences (CLACSO) Latin America & the Caribbean, Argentina
Sanchez, Barbara	Vienna University of Technology, Austria
Strøm, Tanja	Oslo Metropolitan University, Norway
van Leeuwen, Thed	Centre for Science and Technology Studies, Leiden University, The Netherlands
von Schomberg, René	European Commission and TU Darmstadt, Belgium

## Appendix B. List of ON-MERRIT contributors

**Table:**

Angela Fessl	Know-Center and TU Graz, Austria
Anja Rainer	Know-Center, Austria
Antónia Correia	University of Minho, Portugal
Bernhard Wieser	TU Graz, Austria
Birgit Schmidt	University of Göttingen and SUB Göttingen, Germany
David Pride	The Open University, UK
Eloy Rodrigues	University of Minho, Portugal
Ilaria Fava	University of Göttingen and SUB Göttingen, Germany
Ilire Hasani-Mavriqi	Know-Center and TU Graz, Austria
Katherina Maitz	Know-Center, Austria
Najko Jahn	University of Göttingen and SUB Göttingen, Germany
Nancy Pontika	The Open University, UK
Nicki Lisa Cole	Know-Center and TU Graz, Austria
Pedro Príncipe	University of Minho, Portugal
Petr Knoth (Scientific Coordinator)	The Open University, UK
Stefan Reichmann	TU Graz, Austria
Thomas Klebel	Know-Center, Austria
Tony Ross-Hellauer (Coordinator/PI)	Know-Center and TU Graz, Austria
Viktoria Pammer-Schindler	Know-Center and TU Graz, Austria
